# Effects of Streamer Discharge on PM2.5 Containing Endotoxins and Polyaromatic Hydrocarbons and Their Biological Responses In Vitro

**DOI:** 10.3390/ijms232415891

**Published:** 2022-12-14

**Authors:** Akiko Honda, Ken-ichiro Inoue, Shin Tamura, Michitaka Tanaka, Zaoshi Wang, Toshio Tanaka, Seitarou Hirai, Tomoaki Okuda, Kayo Ueda, Hirohisa Takano

**Affiliations:** 1 Graduate School of Global Environmental Studies, Kyoto University, Kyoto 615-8530, Japan; 2 Graduate School of Engineering, Kyoto University, Kyoto 615-8510, Japan; 3 School of Nursing, University of Shizuoka, Shizuoka 422-8526, Japan; 4 Faculty of Pharmaceutical Sciences, Hiroshima International University, Hiroshima 737-0112, Japan; 5 Technology and Innovation Centre, Daikin Industries, Ltd., Osaka 566-8585, Japan; 6 Faculty of Science and Technology, Keio University, Yokohama 223-8522, Japan; 7 Graduate School of Medicine, Hokkaido University, Sapporo 060-8638, Japan

**Keywords:** airway epithelial cells, antigen presenting cells, PM2.5, Asia, inflammation, streamer discharge

## Abstract

Experimental and epidemiological studies have demonstrated that fine particulate matter with a diameter of <2.5 μm (PM2.5) affects both the respiratory and immune systems. However, effective approaches to reduce PM2.5-induced hazardous effects have not been discovered yet. Streamer discharge is a category of plasma discharge in which high-speed electrons collide with oxygen and nitrogen molecules. Although streamer discharge can reportedly eliminate bacteria, molds, chemical substances, and allergens, its ability to decontaminate PM2.5 has not been previously demonstrated. The present study explored whether streamer discharge treatment could reduce PM2.5-induced inflammatory responses by employing an in vitro system. PM2.5 was collected under four conditions (Bangkok (Sep.–Dec.), Bangkok (Dec.–Mar.), Singapore, and Taipei). Airway epithelial cells and antigen-presenting cells exposed to non-treated PM2.5 in several conditions resulted in inflammatory responses. Streamer-discharged PM2.5 (Bangkok (Sep.–Dec.)) decreased the expression of interleukin (IL)-6 and IL-8 compared to non-treated PM2.5. Moreover, composition analysis demonstrated that streamer discharge reduced some compounds, such as endotoxins and polycyclic aromatic hydrocarbons, included in PM2.5 that can elicit inflammatory responses. Streamer discharge treatment can reduce endotoxins, polycyclic aromatic hydrocarbons, and the subsequent inflammatory responses induced by PM2.5 in vitro.

## 1. Introduction

In Asian countries, atmospheric pollution in the form of particulate matter with an aerodynamic diameter of ≤2.5 µm (PM2.5) has become a major cause for concern [1]. Previous experimental and epidemiological studies have revealed that PM2.5 triggers disorders or exacerbates existing diseases in various tissues/organs, including not only the respiratory system (the human body’s initial point of contact with PM2.5) but also the circulatory and immune systems [2,3,4]. In addition, biological pathways mediating the toxic and disease-exacerbating effects of PM2.5 are constantly being discovered [5,6]. Notably, although the chemical composition of PM2.5 greatly varies depending on geographical regions and seasons, the correlations with them and their toxic and disease-exacerbating effects have not been fully elucidated. To thoroughly examine the correlation between the composition of PM2.5 collected from different sites in addition to the effects of these particles on cells, comprehensive in vitro studies need to be performed [7,8].

PM2.5-induced biological effects indeed vary depending on the origin of the particles, and such variations may be explained by the differences among the chemical constituents of PM based on the evaluated sites [9]. Trace metals and endotoxins, for instance, may contribute to PM toxicity [10]. In addition, polycyclic aromatic hydrocarbons (PAHs), polychlorinated dibenzo-p-dioxins (PCDDs), polychlorinated dibenzofurans (PCDFs), and polychlorinated biphenyls (PCBs) are characterized by an established genotoxic potential [11,12] Therefore, the deactivation of the chemical composition of PM2.5 to non-hazardous substances before their entry into the body could prevent/reduce their adverse biological effects.

“Streamer discharge” is a category of plasma discharge in which high-speed electrons collide with oxygen and nitrogen molecules that can cause the oxidative decomposition of bacteria and molds as well as chemical substances and allergens. With respect to the decomposition mechanism associated with streamer technology, a significant streamer discharge affects xenobiotics, decomposing their surface proteins and resulting in destruction through oxidation. The discharge range of the streamer is greater than that of standard plasma discharge (glow discharge). This technology can disseminate various active molecules, which have high oxidation activity generated by their contact with high-speed electrons. Upon reaction, streamer discharge breaks down organic substances into safe molecules, including water and carbon dioxide. Streamer discharge technology has indeed been previously shown to be effective in the inactivation of hazardous materials found in indoor air, such as cedar pollen, influenza virus, norovirus, and toxins and bacteria that can cause food poisoning [13,14,15,16]. Nevertheless, the effects on physical properties and post-exposure biological activities of PM2.5 have to be further elucidated.

In the present study, we aimed to assess the potential improvement of atmospheric air pollution by streamer discharge to prevent PM2.5 from exerting adverse effects on human health. The inflammatory responses of airway epithelial cells and immunocompetent cells to PM2.5 samples collected under different conditions and the in vitro effects of streamer discharge treatment on inflammatory responses or oxidative stress were examined for the purposes of the present study.

## 2. Results

### 2.1. Various PM2.5 Samples Caused Proinflammatory Responses or Oxidative Stress in BEAS-2B Cells

**Figure 1:**
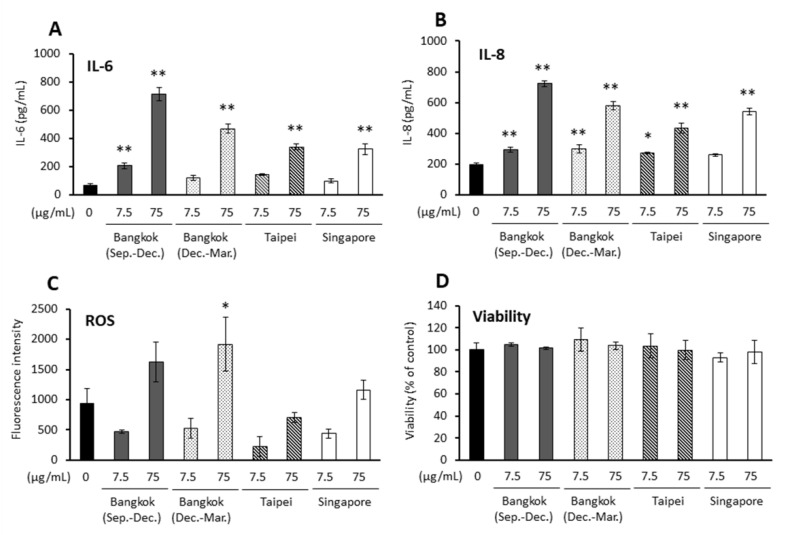
Effects of PM2.5 on proinflammatory responses, oxidative stress, and viability in BEAS-2B cells. (**A**) IL-6 release, (**B**) IL-8 release, (**C**) ROS production, (**D**) cell viability. * *p* < 0.05, ** *p* < 0.01 vs. 0 μg/mL.

PM2.5 was collected under four conditions (Bangkok (Sep.–Dec.), Bangkok (Dec.–Mar.), Singapore, and Taipei). All PM2.5 appeared to induce interleukin (IL)-6 and IL-8 production in a dose-dependent manner in BEAS-2B cells (*p* < 0.01 for IL-6 at 7.5 μg/mL for PM2.5-Bangkok (Sep.–Dec.) and 75 μg/mL for all PM2.5 and for IL-8 at 7.5 μg/mL for PM2.5-Bangkok (Sep.–Dec.)/(Dec.–Mar.) and 75 μg/mL for all PM2.5; P < 0.05 for IL-8 at 7.5 μg/mL for Taipei). On the other hand, PM2.5-Bangkok (Dec.–Mar.) at a dose of 75 μg/mL induced reactive oxygen species (ROS) in BEAS-2B (*p* < 0.05 at 75 μg/mL for PM2.5-Bangkok (Dec.–Mar.)), while no PM2.5 affected cell viability in these cells (Figure 1).

### 2.2. Effects of Several PM2.5 Samples on Activation of Bone Marrow-Derived Antigen Presenting Cells (APCs)

**Figure 2:**
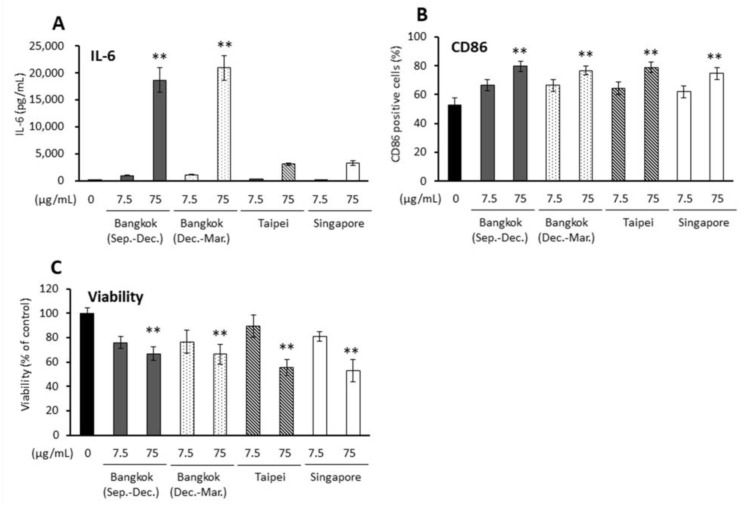
Effects of PM2.5 on proinflammatory responses, CD86 expression, and viability in antigen-presenting cells (APCs). (**A**) IL-6 release, (**B**) CD86 positive cells, (**C**) cell viability. ** *p* < 0.01 vs. 0 μg/mL.

All PM2.5 induced IL-6 production in APCs in a dose-dependent manner (*p* < 0.01 at 75 μg/mL for PM2.5-Bangkok (Sep.–Dec.)/(Dec.–Mar.)). Furthermore, all PM2.5 also activated cluster of differentiation (CD) 86 expression on the APCs and decreased cell viability in a dose-dependent manner (*p* < 0.01 in 75 μg/mL, Figure 2).

### 2.3. Effects of Streamer-Discharged PM2.5 on Proinflammatory Parameters and ROS Production in BEAS-2B Cells

**Figure 3:**
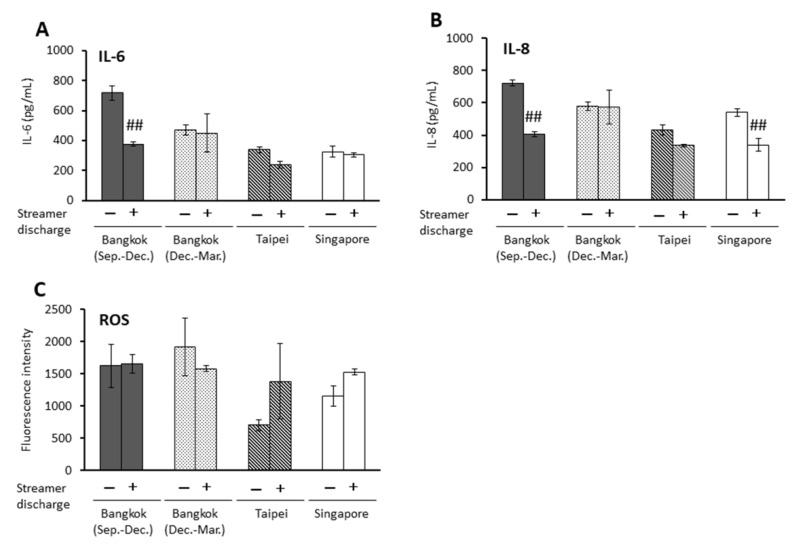
Effects of streamer-discharged PM2.5 at a dose of 75 μg/mL on proinflammatory responses and oxidative stress in BEAS-2B cells. (**A**) IL-6 release, (**B**) IL-8 release, (**C**) ROS production. ## *p* < 0.01 vs. non-treated PM2.5.

Streamer discharge for PM2.5-Bangkok (Sep.–Dec.) at a dose of 75 μg/mL reduced IL-6 and IL-8 significantly (*p* < 0.01), whereas PM2.5-Singapore reduced IL-8 significantly in BEAS-2B cells. Streamer discharge for PM2.5 did not affect induced ROS production by these particles (Figure 3).

### 2.4. Effects of Streamer-Discharged PM2.5 on APCs

**Figure 4:**
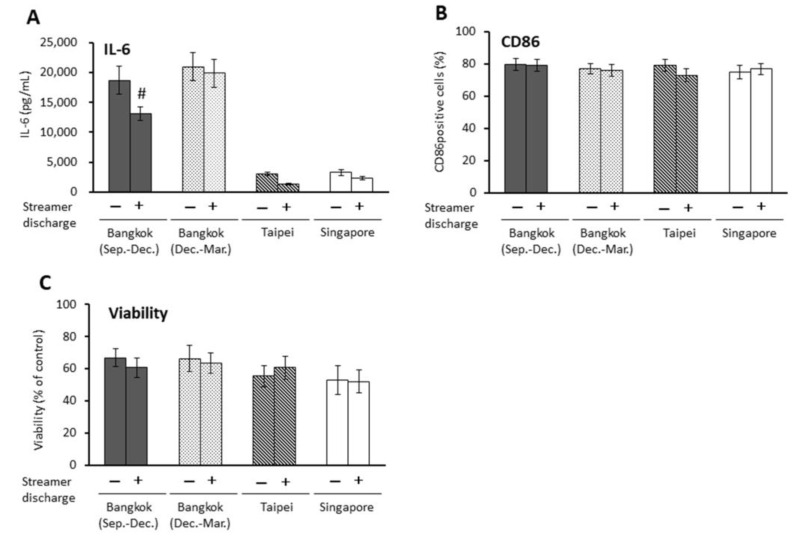
Effects of streamer-discharged PM2.5 at a dose of 75 μg/mL on proinflammatory responses, CD86 expression, and viability in antigen-presenting cells (APCs). (**A**) IL-6 release, (**B**) CD86 positive cells, (**C**) cell viability. # *p* < 0.05 vs. non-treated PM2.5.

Streamer discharge for PM2.5-Bangkok (Sep.–Dec.) at a dose of 75 μg/mL significantly reduced IL-6 (*p* < 0.01), while it did not affect other parameters (Figure 4).

### 2.5. Components in Non-Treated PM2.5 and Streamer-Discharged PM2.5 

Table 1 demonstrates the composition analysis results of PM2.5 samples collected from Bangkok (Sep.–Dec.) before and after the streamer discharge treatment. Reduced PAHs and endotoxins levels were found in the streamer discharge-treated PM2.5 samples. Among carbon components, the streamer discharge treatment decreased EC1–2 and OC2 and increased OC4. Significant modifications were not detected for metals or other inorganic components.

## 3. Discussion

In the current study, various PM2.5 samples induced exposure concentration-dependent increases in the levels of IL-6, IL-8, and ROS production values in human airway epithelial cells in the absence of streamer discharge. Furthermore, various untreated PM2.5 samples induced an increase in IL-6 production and CD86 expression and a reduction in the cellular activity in mouse bone marrow-derived immunocompetent cells in an exposure concentration-dependent manner. The streamer discharge treatment reduced the proinflammatory cytokine production induced by PM2.5-Bangkok (Sep.–Dec.) samples. Furthermore, the results of the thorough chemical composition analysis of the samples collected in Bangkok (Sep.–Dec.) revealed reduced levels of PAHs and endotoxins in the streamer discharge-treated PM2.5 samples.

The biological effects of PM2.5 samples collected in Asian countries have been previously extensively described in epidemiological reports. The severity of atmospheric pollution in Bangkok [17], Taipei [18], and Singapore [19], in which PM2.5 samples were collected for this study, has indeed increased, requiring urgent countermeasures. Nevertheless, PM2.5 samples in various regions have different characteristics, and such variations can be reflected in variations in their toxicity and adverse effects on existing diseases. For the purposes of the present study, PM2.5 samples collected in different geographical regions were initially characterized with respect to their in vitro exposure effects in airway epithelial cells and bone marrow-derived immunocompetent cells. The results of these experiments demonstrated that airway epithelial cells and antigen-presenting cells exposed to non-treated PM2.5 collected in several conditions (sites and seasons) showed inflammatory/activated responses, such as IL-6 and IL-8 production/release and CD86 expression on the cell surface with an overall trend. In addition, PM2.5-Bangkok (Dec.–Mar.) elevated the ROS production value at a dose of 75 μg/mL in airway epithelial cells. The current study thus revealed that PM collected at different sites in southern Asia possesses proinflammatory properties, similar to other PM previously described [20]. PM2.5 collected from Taipei and Singapore induced lower ROS levels (BEAS-2B) and IL-6 (APCs) than the other PM2.5 treatment groups. The content of ROS-producing substances, such as Fe and quinone [21,22], and proinflammatory substances, such as endotoxins [23], may be low in PM2.5 collected in Taipei and Singapore. In any case, due to the number of samples collected in the present study, component analysis was not possible. Detailed differences in the responses of inflammatory/immune cells and/or in vivo models between these particles, however, require further investigation. Moreover, additional studies focused on the relationship between detailed characterization in comprehensive analyses of each PM and the activation of several cells are also required.

Streamer-discharged PM2.5 at a dose of 75 µg/mL reduced IL-6 and IL-8 expression compared to non-treated PM2.5. Exposure to streamer-discharged PM2.5 did not significantly affect ROS production in BEAS-2B cells or CD86 expression and viability in APCs. Streamer-discharged PM2.5-Bangkok (Sep.–Dec.) in particular exhibited a significant reduction in these inflammatory cytokines, although it did not affect ROS production by BEAS-2B cells. The reason why streamer-discharged PM2.5 did not significantly affect ROS production may be that PM2.5 Bangkok (Sep.–Dec.) induced proinflammatory responses mainly through a mechanism not mediated by ROS. Therefore, streamer discharge treatment suppressed proinflammatory responses without changing the amount of ROS production. PM2.5-induced inflammatory cytokines could potentially activate ROS-independent NF-κB activation. According to the above, (1) PM2.5 collected from Bangkok (maybe partially Singapore) can induce, at least partially, inflammatory cytokines from airway epithelial cells via an ROS-independent pathway and (2) streamer discharge for these PM2.5 could suppress the production through ROS-independent NF-κB inactivation and/or other unexplored mechanisms. Additional studies targeting upstream of these cytokines are thus required.

By contrast, a thorough study showed that streamer discharge reduced some compounds, such as endotoxins and PAH. Endotoxin is known to induce inflammatory cytokines, including IL-6 and IL-8. In previous studies, endotoxin has been thought to play an important role in particulate-induced inflammatory responses [24,25,26]. In addition, PAHs can induce IL-6 and IL-8 production through AhR-dependent or independent pathways, while some PAHs containing DEP reportedly promote IL-8 production via NFκB and p38 MAPK pathways in vitro [27]. These components may have thus partly contributed to the reduction of inflammatory markers, such as IL-6 and IL-8, in this study. Furthermore, since the change in EC/OC in PM2.5 induced by streamer discharge might be associated with this phenomenon, analysis of the EC/OC may partially resolve the puzzle in the future.

PAHs and microbial components in particulate matter contribute to inflammatory responses [28,29,30]. Therefore, it is possible that the composition of PM2.5 collected from Bangkok (Dec.–Mar.) and Taipei, especially the concentration of the PAH compounds and endotoxin decreased by streamer discharge, as shown in Table 1, is different from that of PM2.5 from Bangkok (Sep.–Dec.). Indeed, we previously observed that the endotoxin and PAH levels in Bangkok were higher than those in Taipei, although the collection period was different from that of the present study [31]. In Singapore, although the amount was low, the components that were deactivated by streamer discharge may have been contained. Alternatively, it has been reported that metal concentrations in particulate matter are the highest in the cool season (February 2018) among the cool, hot, and rainy seasons (between Feb. and Nov. 2018) in Bangkok. Metals that are less affected by streamer discharge may induce inflammatory reactions and inflammation-associated pyroptosis in PM2.5 collected in Bangkok (Dec.–Mar.) [32,33]. Nevertheless, further detailed analytic research is required to evaluate vital components controlling PM-induced inflammatory response by streamer discharge.

In summary, exposure of airway epithelial cells and APCs to PM2.5 collected from several conditions assisted cellular damage and the activation of inflammatory responses. In addition, streamer discharge partially inhibited this effect. These results provide evidence that the collected PM2.5 elicits inflammatory responses, whereas streamer discharge suppresses the hazardous effects of PM2.5. In particular, streamer discharge can suppress the re-scattering of harmful substances from filters if introduced into the filters of air conditioning systems. Moreover, streamer discharge can improve the safety of workers when replacing the filter. However, further studies are still necessary to elucidate these benefits and to verify that streamer discharge can serve as a promising option for xenobiotic reduction technologies.

## 4. Materials and Methods

### 4.1. PM2.5 Collection

PM2.5 collection was performed using a high-volume PM sampler, the virtual impactor, and the cyclone technique with no filter or extraction process [34]. PM2.5 was collected during different seasons as follows: in Bangkok between September and December 2017 (autumn); in Bangkok between December 2017 and March 2018 (winter); in Taipei between February and May 2017; and in Singapore between June and August 2017. Upon sampling, particles in amber bottles were collected using a stainless spatula.

### 4.2. Cell Culture

The BEAS-2B cell line, which is derived from human bronchial epithelial cells and transformed by an adenovirus 12-SV40 hybrid virus and is not a cancerous phenotype, was purchased from the European Collection of Cell Cultures (Salisbury, Wiltshire, UK). Airway epithelial cells were seeded in 96-well or 12-well collagen I-coated plates and incubated for 72 h to reach semi-confluence in the serum-free medium LHC-9 (Life technologies, Carlsbad, CA, USA) at 37 °C in a humidified atmosphere with 5% CO_2_.

Bone marrow-derived antigen-presenting cells (APCs) were prepared as follows. Ten-week-old SPF NC/NgaTndCrlj male mice were purchased from Charles River Japan (Osaka, Japan). They were housed in an animal facility that was maintained at 24–26 °C with a 12-h light/dark cycle under conventional conditions. The procedures of all animal studies were approved by the Animal Research Committee at Kyoto University. Mice were sacrificed by cervical dislocation and exsanguinated from the cut abdominal aorta and vein. The red blood cells in the bone marrow were lysed with BD PharmLyse (Becton Dickinson, Lincoln Park, NJ, USA). APCs were differentiated using an R10 medium containing 20 ng/mL granulocyte–macrophage colony-stimulating factor (GM-CSF). The differentiated APCs were centrifuged at 400× g for 5 min at 20 °C and then resuspended in a fresh medium. The number of viable cells was determined by the trypan blue exclusion method.

### 4.3. Treatment of PM2.5 with Streamer Discharge

For treatment with streamer discharge, PM2.5 was placed 10 cm from the electrode in the chamber (520 mm × 520 mm × 430 mm) and was treated with active species generated by streamer discharge (5.6 kV × 55 μA) for 140 h. In the preliminary experiment, ambient PM2.5 collected from Japan was treated by streamer discharge for 48 h (2 days) and 184 h (approximately 7 days). Ambient PM2.5 significantly increased IL-6 release compared to the control (0 μg/mL) in BEAS-2B. Streamer discharge decreased IL-6 release in a time-dependent manner. Therefore, PM2.5 was treated by streamer discharge for 140 h in the present study.

### 4.4. Experimental Protocol

After airway epithelial cells were grown to semi-confluence in LHC-9; cells were exposed to PM2.5 at a dose of 7.5 or 75 μg/mL for 3 or 24 h. The cell viability, cytokine production, and ROS generation were examined through WST-1 assay, enzyme-linked immunosorbent assay (ELISA), and 5-(and-6)-chloromethyl-2′,7′-dichlorodihydrofluorescein diacetate, acetyl ester CM-H_2_DCFDA fluorescent probe, respectively.

APCs were exposed to PM2.5 at a dose of 7.5 or 75 μg/mL for 24 h. The cell viability and cytokine and cluster of differentiation (CD) 86 protein expression on the cell surface were evaluated by a fluorescence-activated cell sorter (FACS).

### 4.5. Cell Viability

After exposure to PM2.5 for 24 h, cell viability was assessed by the WST-1 assay using the Premix WST-1 Cell Proliferation Assay System (TaKaRa Bio Inc., Shiga, Japan). In brief, WST-1 reagent was added to each well of a 96-well plate and mixed well by gently rocking the plate. Airway epithelial cells and APCs were incubated with WST-1 reagent at 37 °C for 3 h and 30 min, respectively. After incubation, absorbance was measured on an iMarkMicroplate Absorbance Reader (Bio-Rad Laboratories, Hercules, CA, USA) at a wavelength of 450 nm with a reference wavelength of 630 nm. Results are expressed as the percentage of viable cells over the untreated cells.

### 4.6. Cytokines in the Culture Supernatants

After exposure to PM2.5, the cell culture medium was harvested and centrifuged to remove floating cells. The final supernatants were stored at −80 °C until analysis. The levels of IL-6 and IL-8 (Thermo Scientific, Waltham, Massachusetts, USA) in the culture medium were measured by ELISA according to the manufacturer’s instructions. Absorbance was measured on an iMark Microplate Absorbance Reader at a wavelength of 450 nm with a reference wavelength of 550 nm. The detection limits of the IL-6 and IL-8 assay in airway epithelial cells were lower than 2.9 and 0.49 pg/mL, respectively. The detection limit of the IL-6 assay in APCs was lower than 6.6 pg/mL.

### 4.7. Reactive oxygen Species Generation

For this assay, a CM-H_2_DCFDA fluorescent probe was employed to measure the intracellular reactive oxygen species (ROS) generation. The fluorescence intensity during 0–3 h (excitation 485 nm, emission 530 nm) was then determined.

### 4.8. Fluorescence-Activated Cell Sorting

For Fluorescence-Activated Cell Sorting (FACS) analysis, the following monoclonal antibodies were used: Mouse BD Fc Block™ purified anti-mouse CD16/CD32 (Becton Dickinson), CD86 (GL-1, PE-conjugated, Becton Dickinson), Rat IgG2a, and κ Isotype Control (R35-95, PE-conjugated, Becton Dickinson). After exposure, the cells were resuspended in 50 µL phosphate-buffered saline with 0.3% bovine serum albumin and 0.05% sodium azide (Wako Pure Chemical Industries, Osaka, Japan) and incubated with 1 µg of each antibody for 45 min at 4 °C. After incubation, cells were washed, and their fluorescence was measured using a FACSCalibur (Becton Dickinson). For each sample, fluorescence data from 10,000 cells were collected; positive cells were expressed as % events, or mean fluorescence intensity was calculated.

### 4.9. Characterization of PM2.5

Bangkok autumn and Bangkok winter PM2.5 samples were utilized in the presence and absence of streamer discharge treatment to examine the effects of the streamer discharge on PM2.5 components. According to the “measurement manual for atmospheric particulate matter (PM2.5) components” of the Ministry of the Environment, ion chromatography, Inductively-Coupled Plasma Mass Spectrometry (ICP/MS), thermal optical reflectance, and GS/MS methods were employed in order to quantify water-soluble ion components, metal components, carbon components (EC/OC), and PAHs, respectively, in PM2.5 samples. The levels of endotoxins as inflammatory substances of biological origin were also measured (contractors: Japan Food Research Laboratories (Tokyo, Japan), Japan Environmental Sanitation Center (Kanagawa, Japan), Sumika Chemical Analysis Service, Ltd. (Osaka, Japan)).

### 4.10. Statistical Analysis

Data are presented as means ± standard deviation for each experimental group (n = 3–4). The significance of the variation among PM2.5-exposed groups and the 0 μg/mL group was examined using one-way ANOVA with the Dunnett multiple comparison test. The significance of variation between the streamer-discharged group and the non-treated group was examined using a two-way ANOVA analysis. Differences among groups were assessed using the Tukey multiple comparison test (Excel Statistics 2012, Social Survey Research Information Co., Ltd. Tokyo, Japan). A *p*-value lower than 0.05 indicated statistical significance.

## Figures and Tables

**Table 1:** Composition analysis of PM2.5 collected from Bangkok (Sep.–Dec.) before and after the streamer discharge treatment.

	Before Streamer Discharge	After Streamer Discharge	Rate of Change (%)
***Endotoxin* (EU/mg)**	7.560	4.448	−41.2
***PAH* (ng/mg)**			
Pyrene	0.74	0.37	−50.0
Fluoranthene	0.58	0.29	−50.0
Benz[a]anthracene	0.21	0.1	−52.4
Chrysene	1.1	0.5	−54.5
Benzo[b]fluoranthene	1.2	0.45	−62.5
Benzo[k]fluoranthene	0.33	0.15	−54.5
Benzo[a]pyrene	0.33	0.18	−45.5
Benzo[g,h,i]perylene	1.3	0.61	−53.1
Indeno[1,2,3-c,d]pyrene	0.87	0.37	−57.5
Dibenz[a,h]anthracene	0.08	0.04	−50.0
***Carbon* (ngC/mg)**			
OC1	N.D.	N.D.	N.D.
OC2	20,000	16,000	−20.0
OC3	65,000	66,000	1.5
OC4	23,000	37,000	60.9
EC1	73,000	57,000	−21.9
EC2	93,000	79,000	−15.1
EC3	6000	6200	3.3
***Metals and Other Inorganic Components* (ng/mg)**			
Na	28,000	27,000	−3.6
Al	42,000	38,000	−9.5
K	12,000	12,000	0.0
Ca	33,000	33,000	0.0
Sc	21	20	−4.8
Ti	1300	1300	0.0
V	140	140	0.0
Cr	3400	3500	2.9
Mn	720	690	−4.2
Fe	28,000	27,000	−3.6
Co	29	30	3.4
Ni	1000	1100	10.0
Cu	350	360	2.9
Zn	2700	2500	−7.4
As	69	71	2.9
Se	48	44	−8.3
Rb	48	46	−4.2
Mo	42	41	−2.4
Sb	66	68	3.0
Cs	3.3	3.2	−3.0
Ba	410	410	0.0
La	12	12	0.0
Ce	19	18	−5.3
Sm	0.99	N.D.	N.D.
Hf	1.9	1.3	−31.6
W	77	69	−10.4
Ta	1	0.93	−7.0
Th	2.8	2.4	−14.3
Pb	420	440	4.8
Cd	12	11	−8.3
Cl^−^	4200	3900	−7.1
NO_3_^−^	37,000	36,000	−2.7
SO_4_^2−^	140,000	140,000	0.0
Na^+^	26,000	25,000	−3.8
NH_4_^+^	7600	7800	2.6
K^+^	7500	7600	1.3
Mg^2+^	4000	3800	−5.0
Ca^2+^	32,000	33,000	3.1

N.D.: not detected.

## Data Availability

The data presented in this study are available upon request from the corresponding author. The data are not publicly available due to privacy.
